# Differences in the upslope of the precordial body surface ECG T wave reflect right to left dispersion of repolarization in the intact human heart

**DOI:** 10.1016/j.hrthm.2018.12.006

**Published:** 2019-06

**Authors:** Neil T. Srinivasan, Michele Orini, Rui Providencia, Ron Simon, Martin Lowe, Oliver R. Segal, Anthony W. Chow, Richard J. Schilling, Ross J. Hunter, Peter Taggart, Pier D. Lambiase

**Affiliations:** ∗ Department of Cardiac Electrophysiology, The Barts Heart Center, St Bartholomew’s Hospital, London, United Kingdom; † Institute of Cardiovascular Science, University College London, London, United Kingdom; ‡ Department of Mechanical Engineering, University College London, London, United Kingdom

**Keywords:** Body surface electrocardiogram, Dispersion of repolarization, Surface electrocardiogram, Tpeak-Tend, T wave, T-wave genesis, Ventricular repolarization

## Abstract

**Background:**

The relationship between the surface electrocardiogram (ECG) T wave to intracardiac repolarization is poorly understood.

**Objective:**

The purpose of this study was to examine the association between intracardiac ventricular repolarization and the T wave on the body surface ECG (SECG_TW_).

**Methods:**

Ten patients with a normal heart (age 35 ± 15 years; 6 men) were studied. Decapolar electrophysiological catheters were placed in the right ventricle (RV) and lateral left ventricle (LV) to record in an apicobasal orientation and in the lateral LV branch of the coronary sinus (CS) for transmural recording. Each catheter (CS, LV, RV) was sequentially paced using an S1–S2 restitution protocol. Intracardiac repolarization time and apicobasal, RV–LV, and transmural repolarization dispersion were correlated with the SECG_TW_, and a total of 23,946 T waves analyzed.

**Results:**

RV endocardial repolarization occurred on the upslope of lead V_1_, V_2_, and V_3_ SECG_TW_, with sensitivity of 0.89, 0.91, and 0.84 and specificity of 0.67, 0.68, and 0.65, respectively. LV basal endocardial, epicardial, and mid-endocardial repolarization occurred on the upslope of leads V_6_ and I, with sensitivity of 0.79 and 0.8 and specificity of 0.66 and 0.67, respectively. Differences between the end of the upslope in V_1_, V_2_, and V_3_ vs V_6_ strongly correlated with right to left dispersion of repolarization (intraclass correlation coefficient 0.81, 0.83, and 0.85, respectively; *P* <.001). Poor association between the T wave and apicobasal and transmural dispersion of repolarization was seen.

**Conclusion:**

The precordial SECG_TW_ reflects regional repolarization differences between right and left heart. These findings have important implications for accurately identifying biomarkers of arrhythmogenic risk in disease.

## Introduction

The relationship between intracardiac repolarization of the intact human heart and the surface electrogram T wave (SECG_TW_) is poorly understood. Several markers of repolarization, including QT interval,^1^ JT interval,^2^ and Tpeak-Tend (TpTe),^3^ have been associated with an increased risk of cardiac events, but their relationship to local intracardiac repolarization is poorly understood. Although T waves recorded directly on the intracardiac surface can accurately determine local repolarization time (RT),^4^, ^5^ the SECG_TW_ is thought to represent a far-field recording displaying a summary of repolarization of the entire heart.^6^ Hence, there is uncertainty as to what SECG_TW_ markers represent within the heart.^7^, ^8^, ^9^, ^10^

The normal SECG_TW_ is upright in almost all leads and is concordant to the QRS complex. Yet at the cellular level, depolarization and repolarization reflect current flow in opposite directions. It is hypothesized that in order for the SECG_TW_ to be concordant, waves of depolarization and repolarization must travel in opposite directions.^11^ Several studies have demonstrated opposing depolarizing and repolarizing apicobasal wavefronts.^12^, ^13^, ^14^ Other studies have demonstrated a transmural repolarization gradient^15^, ^16^ and have suggested that TpTe represents transmural dispersion of repolarization.^16^ However, the repolarization sequence of the intact human ventricle is related to the sequence of activation,^17^, ^18^ and this may impart changes on the SECG_TW_.

This study aimed to examine the association between intracardiac ventricular repolarization in the intact human heart and the SECG_TW_, at varying cycle lengths and activation wavefronts, in order to better understand the genesis of the SECG_TW_ and examine the extent to which it represents local intracardiac repolarization.

## Methods

### Patient demographics

Ten patients (mean age 35 ± 15 years; 6 men) with structurally normal hearts undergoing diagnostic electrophysiological study were enrolled. The study was approved by the local ethics committee and conformed to the Declaration of Helsinki. All patients gave informed consent.

### Intracardiac recording and surface T-wave assessment

**Figure 1:**
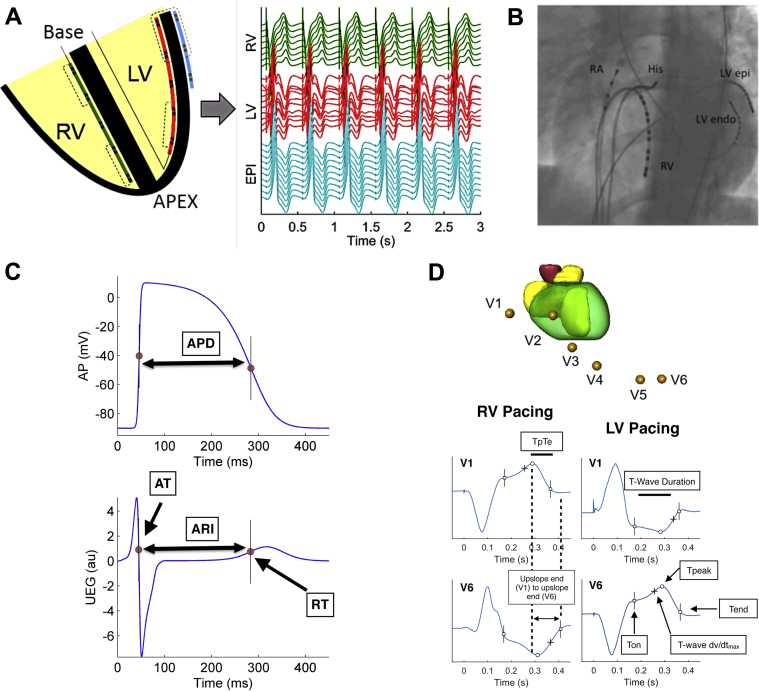
Orientation of catheters in the heart and signal analysis. **A:** Schematic diagram showing positioning of catheters in an apicobasal orientation in the left ventricular (LV) endocardium (LVendo) and right ventricular (RV) endocardium, and transmurally across the lateral base of the LV epicardium (LVepi) via the coronary sinus, with corresponding unipolar electrograms recorded. **B:** Catheter positions were checked by fluoroscopy to ensure adequate apicobasal and transmural apposition. **C:** The Wyatt method was used to analyze repolarization time, with activation time (AT) measured as dv/dt_min_ of the QRS and repolarization time (RT) as dv/dt_max_ of the unipolar intracardiac electrogram (UEG) T wave. Activation recovery interval (ARI = RT – AT) was measured as a surrogate marker of action potential duration (APD). **D:** Schematic diagram of a single beat of the precordial ECG recorded in a patient during RV and LV pacing. Surface ECG markers including T-wave onset (Ton), Tpeak, and Tend were calculated for comparison across all leads during each S2 beat, allowing analysis of T-wave duration, Tpeak-Tend (TpTe), as well as differences across all leads in a single beat of earliest vs latest T-wave upslope end and earliest Ton to latest Tend. AP = action potential; au = arbitrary unit; EPI = epicardium; RA = right atrium.

Our methodology has been described previously^18^ and in the Supplemental Methods. In brief, decapolar catheters were placed in the right ventricle (RV) and lateral wall of the left ventricle (LV) for recording in an apicobasal orientation and the epicardium of the LV (LV_epi_) via the lateral cardiac vein of the coronary sinus (CS) for recording transmurally across the LV wall (Figures 1A and 1B). This configuration allowed us to assess ventricular repolarization across the apicobasal, LV–RV, and endo–epi axes. Restitution curves were performed by pacing in 3 separate regions within the heart: RV apex, LV_endo_ at the base, and LV_epi_ at the base (for further details see the Supplemental Methods).

### Data analysis

At each S2 interval for every pacing location (RV, LV, and CS), SECG_TW_ markers (Figure 1D) were assessed and compared with simultaneously recorded unipolar intracardiac repolarization times (UEGMRT) in the LV and RV (Figure 1D). All 12 leads of the SECG (unipolar and bipolar) were analyzed to enable understanding of the relationship between intracardiac repolarization and the clinical SECG_TW_. In the unipolar contact electrograms, activation time (AT) and RT were measured at the minimum of the first derivative, min(dV/dt), of the signal within the depolarization phase and at the maximum of the first derivative, max(dV/dt), of the signal during the T wave, respectively (Figure 1C). The activation recovery interval, a standard surrogate of local action potential duration, was measured as RT – AT (Figure 1C). Dispersion of repolarization was computed as the interval between minimum and maximum RT. For every beat, ECG marker differences across individual leads and between leads were assessed for comparison of repolarization dispersion in the major anatomic axis. Apicobasal repolarization differences (measured as the largest difference between apex minus base RTs with the heart), transmural dispersion of repolarization of the LV basal wall (measured as the largest difference between endocardial minus epicardial RT), and right to left repolarization dispersion (measured as the largest difference of right and left RTs).

SECG_TW_ was analyzed for time of onset of the T wave (Ton), peak of the T wave (Tpeak), and end of the T wave (Tend), in every ECG lead in every patient (Figure 1D) at every cycle length. Tpeak was identified as the maximum of upright and minimum of inverted T waves, whereas Ton was localized as the local inflection point at the onset of the T wave. Tend was calculated using the tangent method as the intersection between the tangent to the latest flank of the T wave and baseline. This allowed comparison of T-wave duration, Tpeak, earliest Ton to latest Tend, and differences between the upslope ends between different leads and intracardiac repolarization during a single beat across all 12 ECG leads and their association of repolarization dispersion in the major anatomic axes.

In this study, T-wave upslope refers to the ascending flank of the T wave, and “upslope end” refers to the end of the ascending flank of a T wave, which corresponds to Tpeak in upright T waves and to Tend in inverted T waves. The interval between early and late upslope end was used as an estimate of repolarization dispersion. In total, 23,946 individual SECG_TW_ were analyzed and compared to regional UEGMRT to assess the association of SECG_TW_ to UEGMRT regardless of pacing cycle length and activation wavefront. From 60–70 restitution S2 points were collected per drive train, and this was performed in 3 different revisions of the heart in 20 patients, with 12 surface ECG leads connected to the patients, for a total of 24,482 individual S2 T waves, of which 536 were discarded because of ECG noise. All markers were measured with the semi-automatic bespoke MatLab interface as in previous studies^18^ and manually corrected if needed.

### Statistical analysis

Comparisons between measured intracardiac RT and SECG markers were assessed using a paired *T* test. Measurement similarity between SECG T-wave markers and the intracardiac T wave were assessed by calculating the intraclass correlation coefficient (ICC), using a 2-way mixed model of absolute agreement. The relationship between the upslope of the T wave on the SECG, regardless of polarity, and regional intracardiac RT was assessed using sensitivity and specificity analysis. The relationship between the dispersion of repolarization and measures within the ECG T wave were assessed using ICC and R^2^ of linear regression. *P* ≤.05 was considered statistically significant. Statistical analysis was performed using R statistical computing software (version 3.2.2).^19^

## Results

### Polarity of surface ECG T wave in relation to intracardiac electrogram

**Figure 2:**
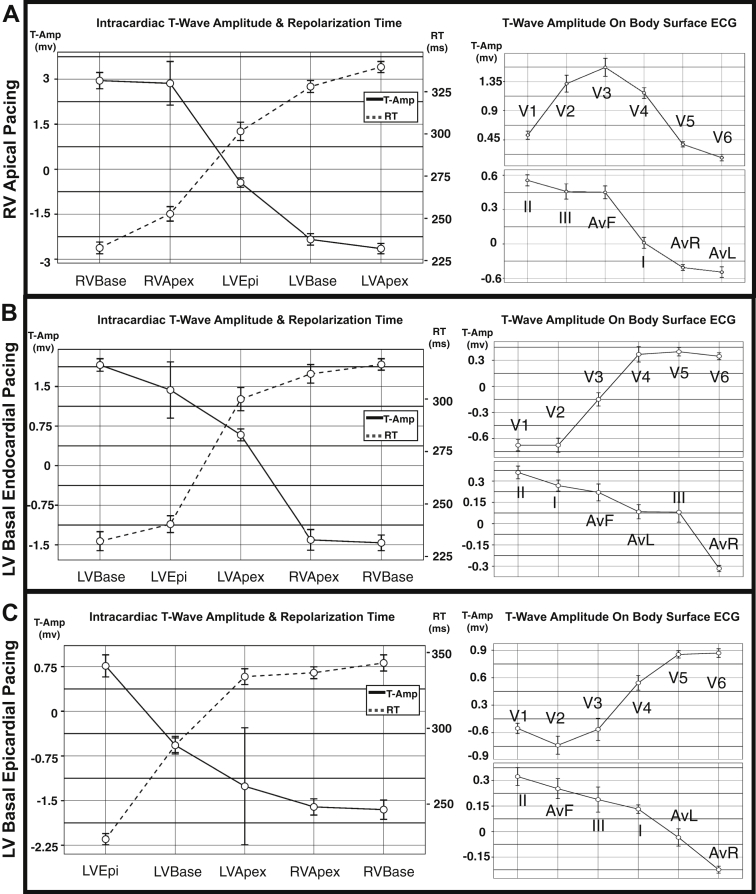
Unipolar T-wave amplitude regionally within the heart in relation to local repolarization time and body-surface 12-lead ECG amplitude. Rows depict mean and 95% confidence interval data for restitution pacing during right ventricular apical pacing **(A)**, left ventricular basal endocardial pacing **(B)**, and left ventricular basal epicardial **(C)** pacing. Epi = epicardium; LV = left ventricle; RT = repolarization time; RV = right ventricle; T-Amp = T-wave amplitude.

The amplitude and polarity of the precordial SECG_TW_ depend on the repolarization sequence within the myocardium (Figure 2).

During RV pacing (Figure 2A), there is trend toward a positive SECG_TW_ in leads V_1_–V_4_ and a negative SECG_TW_ in V_5_–V_6_. This matches the distribution within the myocardium (Figure 2A), where early repolarizing sites (RV base and apex) have a positive EGM_TW_, whereas late repolarizing sites (LV basal epicardium, basal endocardium, and apex) have low-amplitude or negative EGM_TW_. During LV pacing at the basal endocardium (Figure 2B) and basal epicardium (Figure 2B), the opposite pattern in precordial lead SECG_TW_ polarity is observed, with a negative amplitude in the RV leads (V_1_–V_2_) and a positive amplitude in the LV leads (V_5_–V_6_). This again corresponds to the pattern of regional intracardiac repolarization (Figures 2B and 2C).

The limb leads on the SECG_TW_ showed no consistent pattern in SECG_TW_ polarity, despite the change in repolarization dispersion, in the apicobasal, transmural LV, and RV to LV orientation (Figure 2). Supplemental Table 1 and Supplemental Figure 1 show the intraclass correlation (ICC) between regional EGM_TW_ and SECG_TW_ amplitude, including all pacing sites, through the whole of the restitution protocol in all patients. Strong agreement is demonstrated between V_1_ and V_2_ and the amplitude of EGM_TW_ at the RV base ( ICC 0.78 and 0.61, respectively; *P* <.001). Moderate agreement was demonstrated between V_6_ and the LV base endocardially (ICC 0.3; *P* <.001) and epicardially (ICC 0.28; *P* <.001).

### Relationship of intracardiac repolarization to markers on the SECG

Independently of the pacing site, the earliest Ton in the SECG_TW_ always preceded intracardiac RT (difference between Ton and earliest intracardiac RT = –85 ± 45 ms; *P* <.001), whereas the latest Tend in the SECG_TW_ always followed RT (difference between latest Tend and latest intracardiac RT = 43 ± 25 ms; *P* <.001).

**Figure 3:**
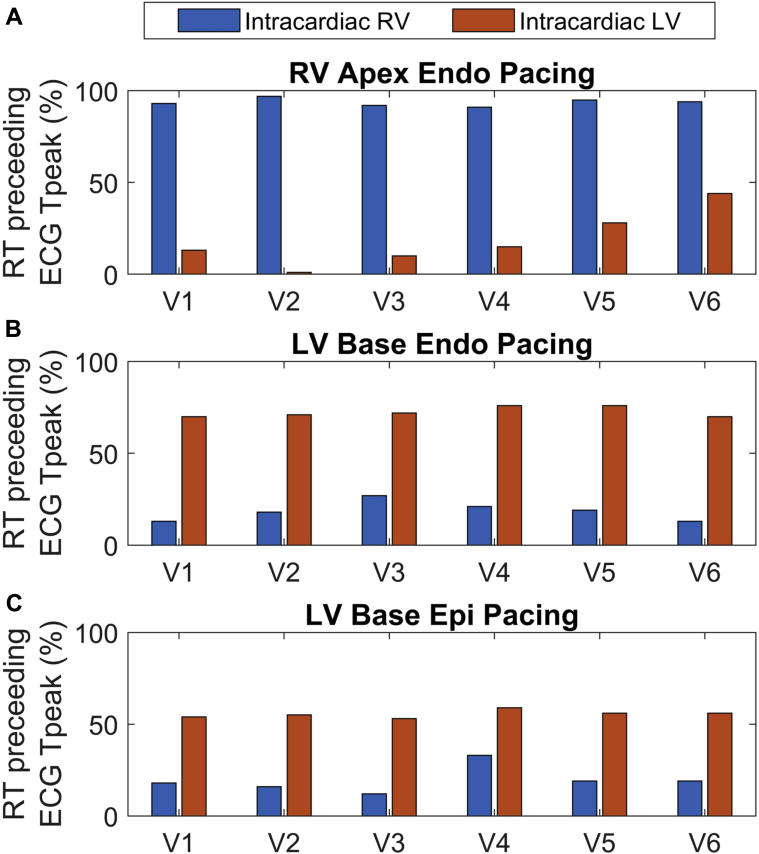
Proportion of right ventricular (RV) and left ventricular (LV) sites that repolarized before Tpeak on the precordial ECG leads, during RV apical endocardial pacing **(A)**, LV basal endocardial pacing **(B)** and LV basal epicardial pacing **(C)**. Endo = endocardial; Epi = epicardium; RT = repolarization time.

The proportion of sites that repolarized before T peak on the SECG showed significant heterogeneity between the LV and RV, based on the location of the pacing site. During RV pacing, a greater proportion of RV sites repolarized before T peak, whereas LV sites repolarized after T peak (Figure 3A and Supplemental Table 2). During LV pacing both endocardially and epicardially, a greater proportion of LV sites repolarized before Tpeak (Figures 3B and 3C, and Supplemental Table 2).

### Relationship between regional intracardiac repolarization to SECG_TW_ upslope

**Figure 4:**
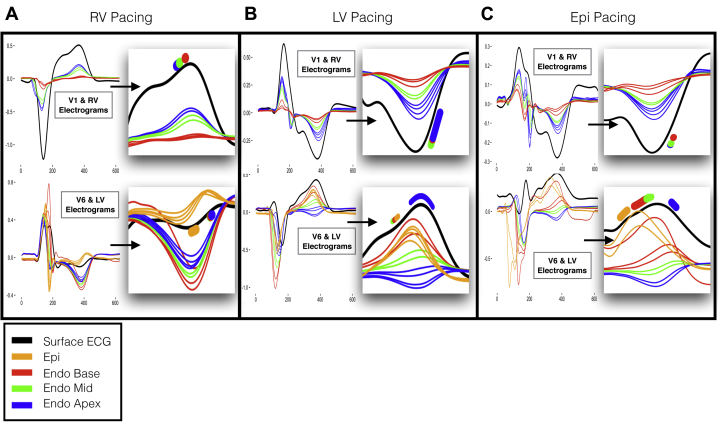
Relationship between repolarization measured in the intracardiac unipolar electrogram and the T wave in leads V_1_ and V_6_ on the surface ECG in a single patient during right ventricular (RV) endocardial **(A)**, left ventricular (LV) endocardial **(B)**, and left ventricular epicardial (Epi) **(C)** pacing. **A–C:** Simultaneously recorded unipolar electrograms from the RV and LV during a single pacing beat. Colors represent the electrograms in the base *(red)*, mid *(green)*, apex *(blue)*, and epicardium *(orange)*, along with the body surface ECG *(black)*. Inset within each panel is focused view of the T wave, with range of measured regional repolarization moments overlaid on the body surface ECG T wave. Endo = endocardial.

Figure 4 shows the relationship between regional intracardiac RT to the morphology of the SECG_TW_ in leads V_1_ and V_6_, in a single beat in 1 patient during pacing from the RV, LV endocardium, and LV epicardium. Consistency between the morphology and polarity of regional EGM_TW_ and the most proximal SECG_TW_ is confirmed. Furthermore, local repolarization within each cardiac region consistently occurred during the upslope of the most proximal SECG_TW_:

• During RV pacing, early RV repolarization occurred within the upslope of SECG_TW_ in V_1_, whereas late LV repolarization occurred within the upslope of SECG_TW_ in V_6_ (Figure 4A, insets).
• During LV endocardial and epicardial pacing, early LV repolarization occurred within the upslope of SECG_TW_ in V_6_, whereas late RV repolarization occurred within the upslope of SECG_TW_ in V_1_ (Figures 4B and 4C, insets).

**Figure 5:**
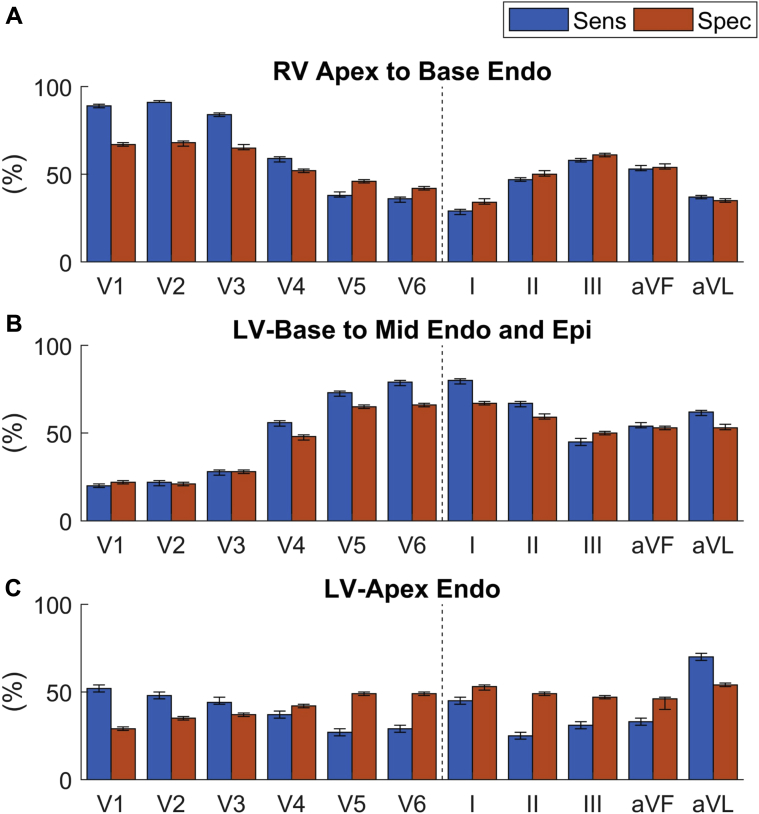
Relationship between sensitivity (Sens) and specificity (Spec) of the T-wave upslope to repolarization time within regions of the heart, endo RV apex to base **(A)** LV endo mid to base and epi **(B)**, LV endo apex **(C)**. Endo = endocardium; Epi = epicardium; LV = left ventricle; RV = right ventricle.

Statistical analysis in all patients, cycle lengths, and pacing sites confirmed this observation (Figure 5 and Supplemental Table 3). RV endocardial RTs, including all measured regions from apex to base, occurred on the SECG_TW_ upslope in V_1_, V_2_, and V_3_, with sensitivity of 0.89, 0.91, and 0.84, and specificity of 0.67, 0.68, and 0.65, respectively. As the precordial SECG markers moved further away from the RV anatomically (V_4_–V_6_), sensitivity and specificity decreased, and the limb leads showed generally poor sensitivity and specificity for repolarization moments in the RV. LV basal endocardial, epicardial, and mid-endocardial regions displayed the opposite phenomenon, with sensitivity of 0.79 and 0.8, and specificity of 0.66 and 0.67 in leads V_6_ and I, respectively, but with decreasing sensitivity and specificity from leads V_5_ to V_1_, and poor sensitivity and specificity in the rest of the limb leads. Finally, LV apical RTs showed poor sensitivity and specificity to the upslope of the SECG, with only aVR showing sensitivity of 0.76 but with poor specificity of 0.52.

### Relationship of SECG_TW_ to dispersion of repolarization in the major anatomic axes

TpTe has previously been reported as a marker of dispersion of repolarization, transmurally in the wedge preparation^16^, ^20^ or globally within the whole heart in animal studies.^10^ We studied the relationship between TpTe, the time difference between the end of the SECG_TW_ upslope across all leads, and the difference between the start and end of the SECG_TW_ in all leads to dispersion of repolarization in the major anatomic axes.

**Figure 6:**
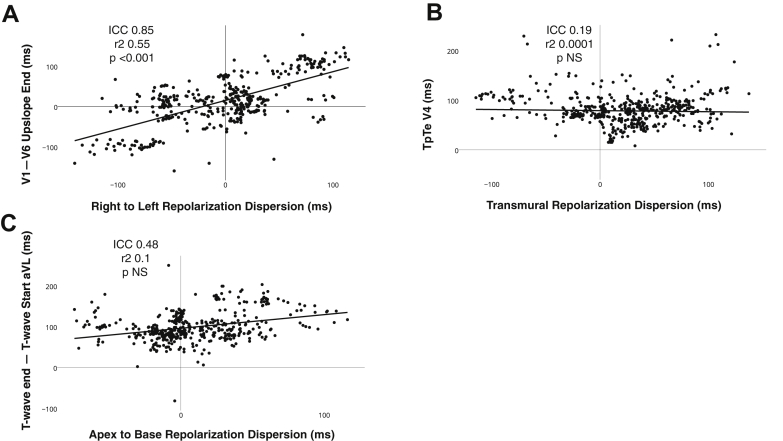
Relationship between dispersion of repolarization in the major anatomic axis and markers on the surface ECG T-wave. **A:** Example of right to left dispersion of repolarization in relation to the difference between the end of the upslope in V_1_ minus the end of the upslope in V_6_. **B:** Example of the best correlation of Tpeak-Tend (TpTe) and transmural dispersion of repolarization, seen in V_4_. **C:** Example of the best correlation between apicobasal dispersion of repolarization and surface ECG markers, seen by measuring the difference between the end and the start of the T wave in lead aVL. ICC = intraclass correlation coefficient.

A strong correlation was seen between right to left dispersion of repolarization and the difference between the end of the SECG_TW_ upslope in lead V_1_ vs V_6_ (ICC 0.81; R = 0.45; *P* <.001), lead V_2_ vs V_6_ (ICC 0.83; R = 0.5; *P* <.001), lead V_3_ vs V_6_ (ICC 0.85; R = 0.55; *P* <.001), V_1_ vs aVL (ICC 0.82; R = 0.5; *P* <.001), V_2_ vs aVL (ICC 0.81; R = 0.42; *P* <.001), and V_3_ vs aVL (ICC 0.83; R = 0.55; *P* <.001) (Figure 6A), regardless of T-wave polarity, pacing site, or cycle length. No strong correlations existed between the difference in the end of the SECG_TW_ upslopes in any other lead and right to left dispersion (best ICC <0.5 for all other variables). No strong correlations existed between the difference between the end SECG_TW_ upslopes and apicobasal (best ICC <0.5 for all measures) or transmural dispersion of repolarization (best ICC <0.5 for all measures).

We were not able to demonstrate any strong correlation between TpTe measured in all 12 ECG leads, particularly in V_4_, V_5_, V_6_, or lead II on the SECG and dispersion of repolarization in the transmural (best ICC <0.2 for all measures), apicobasal (best ICC <0.12 for all measures), or right to left orientations (best ICC <0.22 for all measures) (Figure 6B). Additionally, differences between the start and the end of the T wave and the start and the end of the T-wave upslope did not demonstrate any strong correlation with dispersion of repolarization (Figure 6C) in the right to left axis (best ICC <0.47 for all measures), apicobasal axis (best ICC <0.48 for all measures), and transmural dispersion of repolarization (best ICC <0.23 for all measures).

## Discussion

This is the first study to provide direct correlation between local repolarization in the major anatomic axis and SECG_TW_ in the intact human heart. The main findings are as follows: (1) the amplitude/polarity of the T wave on the precordial leads reflects the polarity of the unipolar signal recorded on the underlying nearby myocardium; (2) local RTs in the RV occur along the upslope of the SECG_TW_ in leads V_1_, V_2_, and V_3_, whereas local repolarization in the LV occurs during the SECG_TW_ upslope in leads V_5_, V_6_, and I; (3) the difference between the end of the T-wave upslope time in V_1_ minus V_6_ provides a good representation of right to left dispersion of repolarization; (4) no strong markers for apicobasal or transmural repolarization differences were seen on the SECG; and (5) TpTe did not correlate with dispersion of repolarization in the right to left, apicobasal, or transmural axis.

### Polarity of the precordial lead SECG_TW_ mirrors the EGM_TW_ of the underlying myocardium

The polarity and upslope of the contact EGM_TW_ are related the local repolarization component of the underlying myocardium.^4^, ^5^, ^21^ The EGM_TW_ is more positive when the repolarization of local tissue is early, biphasic in intermediate RTs, and negative in late depolarizing sites where the rest of the heart has repolarized.^21^ The far-field or whole heart component is represented by the downslope of the EGM_TW_. Our data using the well-validated Wyatt method^4^, ^5^ confirm this finding (Figure 2), with early repolarizing sites having an upright T wave but late repolarizing sites having a negative T wave. The precordial lead SECG_TW_ mirrored the polarity of the T wave in the underlying myocardium, with V_5_ and V_6_ matching the polarity of sites measured in the LV(Supplemental Table 1), whereas V_1_, V_2_, and V_3_ matched the polarity of sites measured in the RV (Figure 2 and Supplemental Table 1). The T waves in the limb leads displayed no pattern in relation to local repolarization, possibly because of their substantial distance from the myocardium, thus representing a far-field electrogram of whole heart repolarization. The lack of correlation between SECG_TW_ polarity and the apex of the LV may highlight that the precordial SECG fails to extend inferiorly enough to cover the local repolarization of the LV apex and the overlap with the RV apex.

### Right and left heart intracardiac RTs occur along the upslope of the precordial SECG_TW_ regardless of polarity

The upslope of V_1_–V_3_ showed good sensitivity to all measured RV repolarization, whereas the upslope of V_5_–V_6_ and I showed good sensitivity to transmural LV basal and LV mid-myocardial repolarization (Supplemental Table 3). These findings were independent of T-wave polarity and activation wavefront. Yamaki et al^22^ previously demonstrated that ventricular AT, measured as the QRS downstroke time on the body surface ECG, closely correlated to directly measured ventricular activation and activation delay in LV hypertrophy. Our finding that regional ECG T-wave upslope correlates with directly measured ventricular RT would be in keeping with these data as repolarization is the electrically opposite phenomenon to depolarization. It has previously been suggested that variations in the transmural gradient across the ventricular wall^20^ may inscribe the morphology of the SECG_TW_, but our data do not support this. Regardless of the transmural gradient (Figures 4B and 4C), repolarization of the base of the LV occurred along the upslope of the V_6_, and this did not alter its polarity. This is perhaps due to the differences between experimental studies and our intact whole human heart studies, in which the influence of the far-field or global myocardial muscle mass has a greater influence on the SECG_TW_.

### SECG_TW_ and dispersion of repolarization in the major anatomic axis

Our data show that differences between the end of the upslope in V_1_, V_2_, and V_3_ vs V_6_/aVL provide excellent correlation to right to left dispersion of repolarization (Figure 6A), regardless of the polarity of the T wave, cycle length, or activation wavefront. Poor correlation existed between TpTe and dispersion of repolarization in the transmural, apicobasal, and right to left axis (Figure 6B). This is in contrast to previous studies^20^ and again reflect differences between local and far-field electrogram components in experimental studies compared to whole heart studies. This highlights the limitation of TpTe in a single SECG_TW_ as a measure of dispersion of repolarization.^23^ Our data suggest that the upslope in the precordial lead SECG_TW_ represents local regional repolarization of the nearby underlying myocardium. Thus, if the SECG T wave is negative, TpTe may reflect a local repolarization component; however, if the T wave is positive, it may represent a difference between the end of a regional repolarization component and the far-field or late repolarization regions within the heart. This may explain Figure 6B, as TpTe is a constant measure of the balance between local and global repolarization.

There was no strong relationship to apicobasal dispersion of repolarization and SECG markers (Figure 6C). Previous work has suggested that T-wave morphology may be inscribed by predominant apicobasal differences in repolarization.^8^ Meijborg et al^24^ suggested that differences in the earliest peak to the latest end of the SECG_TW_ reflect global dispersion of repolarization within the porcine heart, in which apicobasal differences predominate. In the intact human heart, however, differences in repolarization between the thin RV and the large muscle mass of the LV may predominate, reflecting species differences.

### Study limitations

Data were confined to multielectrode unipolar contact catheter recordings in the human heart as opposed to global mapping. This was because it was not possible to use global mapping systems in patients admitted for a minimally invasive human study. In addition, although great care was taken to transmurally oppose the catheters, true transmural recordings like those of wedge preparations or plunge electrode recordings were not possible. We did not assess global measures of TpTe as assessed in some other studies; therefore, comparisons with these studies are not possible.^24^ ECG markers of repolarization have been derived from a standard 12-lead ECG configuration. Future studies may assess the interaction between intracardiac repolarization dynamics and ECG repolarization markers derived from orthogonal leads.

## Conclusion

The upslope of the T wave in the precordial leads on the surface ECG represents regional repolarization within the underlying RV and LV. Differences between the end of the upslope in V_1_–V_3_ vs V_6_/aVL represent right to left dispersion of repolarization. Further assessment of the consistency of this marker in structurally abnormal intact human hearts and its role in risk prediction is needed. There was no correlation between TpTe and dispersion of repolarization in the intact human heart.
