# Supplementary figures and images for: Differences in the upslope of the precordial body surface ECG T wave reflect right to left dispersion of repolarization in the intact human heart
Source: Heart Rhythm. 2019 Jun;16(6):943–51. doi: 10.1016/j.hrthm.2018.12.006 (PMC6546969; doi:10.1016/j.hrthm.2018.12.006)

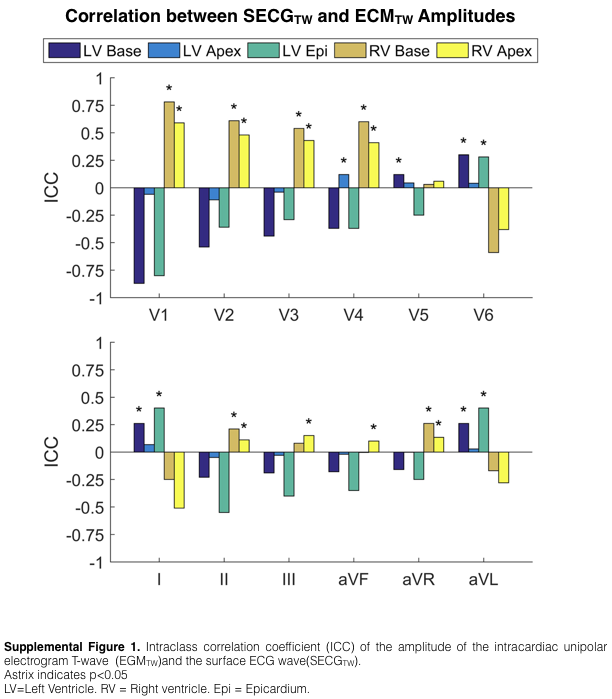

Supplement: Supplemental Figure 1 [file mmc1.docx]
