# Supplementary material for: Differences in the upslope of the precordial body surface ECG T wave reflect right to left dispersion of repolarization in the intact human heart
Source: Heart Rhythm. 2019 Jun;16(6):943–51. doi: 10.1016/j.hrthm.2018.12.006 (PMC6546969; doi:10.1016/j.hrthm.2018.12.006)
Supplement: Supplemental Table 1 [file mmc2.docx]

**Supplemental Table 1.**

Relationship Between The Amplitude of the T-wave on the SECG to the Regional Intracardiac T-wave

|  |  | **LV Base Tamp** | **LV Epi Tamp** | **LV Apex Tamp** | **RV Apex Tamp** | **RV Base Tamp** |
| --- | --- | --- | --- | --- | --- | --- |
| **V1** | **ICC** | -0.87 | -0.80 | -0.06 | **0.59** | **0.78** |
|  | **P-value** | ns | ns | 0.92 | **<0.001** | **<0.001** |
| **V2** | **ICC** | -0.54 | -0.36 | -0.11 | **0.48** | **0.61** |
|  | **P-value** | ns | ns | 0.99 | **<0.001** | **<0.001** |
| **V3** | **ICC** | -0.44 | -0.29 | -0.04 | **0.43** | **0.54** |
|  | **P-value** | ns | ns | 0.83 | **<0.001** | **<0.001** |
| **V4** | **ICC** | -0.37 | -0.37 | **0.12** | **0.41** | **0.6** |
|  | **P-value** | ns | ns | **0.002** | **<0.001** | **<0.001** |
| **V5** | **ICC** | **0.12** | -0.25 | 0.043 | 0.058 | 0.03 |
|  | **P-value** | **0.022** | ns | 0.15 | 0.07 | 0.28 |
| **V6** | **ICC** | **0.30** | **0.28** | 0.04 | -0.38 | -0.59 |
|  | **P-value** | **<0.001** | **<0.001** | 0.17 | ns | ns |
| **I** | **ICC** | **0.26** | **0.40** | 0.068 | -0.51 | -0.25 |
|  | **P-value** | **<0.001** | **<0.001** | 0.06 | ns | ns |
| **II** | **ICC** | -0.23 | -0.55 | -0.05 | **0.11** | 0.21 |
|  | **P-value** | ns | ns | 0.84 | **0.003** | <0.001 |
| **III** | **ICC** | -0.19 | -0.40 | -0.03 | **0.15** | 0.08 |
|  | **P-value** | ns | ns | 0.77 | **<0.001** | 0.07 |
| **aVF** | **ICC** | -0.18 | -0.35 | -0.02 | 0.10 | -0.004 |
|  | **P-value** | ns | ns | 0.72 | 0.005 | 0.53 |
| **aVR** | **ICC** | -0.16 | -0.25 | -0.003 | **0.134** | 0.26 |
|  | **P-value** | ns | ns | 0.53 | **<0.001** | <0.001 |
| **aVL** | **ICC** | **0.26** | **0.4** | 0.028 | -0.28 | -0.17 |
|  | **P-value** | **<0.001** | **<0.001** | 0.26 | ns | ns |
