# Supplementary material for: Differences in the upslope of the precordial body surface ECG T wave reflect right to left dispersion of repolarization in the intact human heart
Source: Heart Rhythm. 2019 Jun;16(6):943–51. doi: 10.1016/j.hrthm.2018.12.006 (PMC6546969; doi:10.1016/j.hrthm.2018.12.006)
Supplement: Supplemental Table 2 [file mmc3.docx]

**Supplemental Table 2.**

Percentage of all recorded sites RV and LV that repolarized before T-peak on the SECG

| **Pacing Site** | **Repolarized sites** | **V1** | **V2** | **V3** | **V4** | **V5** | **V6** | **I** | **II** | **III** | **aVL** | **aVR** | **aVF** |
| --- | --- | --- | --- | --- | --- | --- | --- | --- | --- | --- | --- | --- | --- |
| **RV apex** | RV (%) | 93 | 97 | 92 | 91 | 95 | 94 | 85 | 92 | 93 | 92 | 94 | 93 |
|  | LV (%) | 13 | 1 | 10 | 15 | 28 | 44 | 24 | 20 | 15 | 11 | 31 | 16 |
| **LV endo** | RV (%) | 13 | 18 | 27 | 21 | 19 | 13 | 14 | 28 | 13 | 7 | 16 | 18 |
|  | LV (%) | 70 | 71 | 72 | 76 | 76 | 70 | 76 | 72 | 68 | 92 | 71 | 67 |
| **LV epi** | RV (%) | 18 | 16 | 12 | 33 | 19 | 19 | 20 | 21 | 20 | 11 | 18 | 16 |
|  | LV (%) | 54 | 55 | 53 | 59 | 56 | 56 | 55 | 58 | 57 | 46 | 54 | 58 |
