# Supplementary material for: Differences in the upslope of the precordial body surface ECG T wave reflect right to left dispersion of repolarization in the intact human heart
Source: Heart Rhythm. 2019 Jun;16(6):943–51. doi: 10.1016/j.hrthm.2018.12.006 (PMC6546969; doi:10.1016/j.hrthm.2018.12.006)
Supplement: Supplemental Table 3 [file mmc4.docx]

**Supplemental Table 3.**

Sensitivity and Specificity Of The Upslope Of The SECG To Regional Repolarization Within The Heart

| **Surface ECG Lead Upslope** | **RV Apex to Base (endo)** | | **LV-Base to Mid (endo & epi)** | | **LV-Apex (endo)** | |
| --- | --- | --- | --- | --- | --- | --- |
|  | **Sensitivity** | **Specificity** | **Sensitivity** | **Specificity** | **Sensitivity** | **Specificity** |
| **V1** | **0.89**  **(0.88-0.90)** | 0.67  (0.66-0.68) | 0.20  (0.19-0.21) | 0.22  (0.21-0.23) | 0.52  (0.50-0.54) | 0.29  (0.28-0.30) |
| **V2** | **0.91**  **(0.91-0.92)** | 0.68  (0.66-0.69) | 0.22  (0.20-0.23) | 0.21  (0.20-0.22) | 0.48  (0.46-0.50) | 0.35  (0.34-0.36) |
| **V3** | **0.84**  **(0.83-0.85)** | 0.65  (0.64-0.67) | 0.28  (0.26-0.29) | 0.28  (0.27-0.29) | 0.44  (0.43-0.47) | 0.37  (0.36-0.38) |
| **V4** | 0.59  (0.57-0.60) | 0.52  (0.51-0.53) | 0.56  (0.54-0.57) | 0.48  (0.46-0.49) | 0.37  (0.35-0.39) | 0.42  (0.41-0.43) |
| **V5** | 0.38  (0.37-0.40) | 0.46  (0.45-0.47) | 0.73  (0.71-0.74) | 0.65  (0.64-0.66) | 0.27  (0.25-0.29) | 0.49  (0.48-0.50) |
| **V6** | 0.36  (0.34-0.37) | 0.42  (0.41-0.43) | **0.79**  **(0.77-0.80)** | 0.66  (0.65-0.67) | 0.29  (0.27-0.31) | 0.49  (0.48-0.50) |
| **I** | 0.29  (0.27-0.30) | 0.34  (0.33-0.36) | **0.80**  **(0.78-0.81)** | 0.67  (0.66-0.68) | 0.45  (0.43-0.47) | 0.53  (0.51-0.54) |
| **II** | 0.47  (0.46-0.48) | 0.50  (0.49-0.52) | 0.67  (0.65-0.68) | 0.59  (0.58-0.61) | 0.25  (0.23-0.27) | 0.49  (0.48-0.50) |
| **III** | 0.58  (0.57-0.59) | 0.61  (0.60-0.62) | 0.45  (0.43-0.47) | 0.50  (0.49-0.51) | 0.31  (0.29-0.33) | 0.47  (0.46-0.48) |
| **aVF** | 0.53  (0.52-0.55) | 0.54  (0.53-0.56) | 0.54  (0.53-0.56) | 0.53  (0.52-0.54) | 0.33  (0.31-0.35) | 0.46 (0.45-0.47) |
| **aVL** | 0.37  (0.36-0.38) | 0.35  (0.34-0.36) | 0.62  (0.60-0.63) | 0.53  (0.52-0.55) | 0.70  (0.68-0.72) | 0.54  (0.53-0.55) |
| **aVR** | 0.61  (0.60-0.62) | 0.53  (0.52-0.55) | 0.26  (0.25-0.28) | 0.35  (0.34-0.36) | **0.76**  **(0.74-0.77)** | 0.52  (0.51-0.53) |

*Numbers highlighted in bold indicate a sensitivity >75%*
