# Supplementary material for: Differences in the upslope of the precordial body surface ECG T wave reflect right to left dispersion of repolarization in the intact human heart
Source: Heart Rhythm. 2019 Jun;16(6):943–51. doi: 10.1016/j.hrthm.2018.12.006 (PMC6546969; doi:10.1016/j.hrthm.2018.12.006)
Supplement: Supplemental Methods [file mmc5.docx]

Supplement 1 -- Detailed Methods

Methods

Patient Demographics

Ten patients (mean age was 35 +/- 15yrs 6 Male) with structurally normal hearts who were due to undergo diagnostic electrophysiology study (EPS) for investigation of supraventricular tachycardia (SVT) were enrolled into the study. Studies were performed in the post-absorptive state under minimal conscious sedation. All patients had normal resting electrograms with no evidence of latent preexcitation, normal echocardiograms and normal cardiac examination. All anti-arrhythmic drugs were stopped for 5 days prior to procedure. The study was approved by the local ethics committee and conformed to the declaration of Helsinki. All patients gave informed consent. The research protocol began after a 20-minute period of resting sinus rhythm at the end of clinical EPS


Experimental Protocol

The methodology for our study is previously described elsewhere. Briefly decapolar catheters (Response; St. Jude Medical, Minnetonka, MN) were placed in the right ventricle (RV) and lateral wall of the Left Ventricle (LV) for recording in an apico-basal orientation, and a steerable decapolar catheter (Inquiry; St. Jude Medical, Minnetonka, MN) was placed on the epicardium of the LV (LVepi) via the lateral cardiac vein of the coronary sinus (CS) for recording transmurally across the LV wall (Figure 1. A &B). This configuration allowed us to assess ventricular repolarization across the Apical-Basal, LV-RV and endo-epi axes. Programmed electrical stimulation was performed at a pulse width of 2 ms and stimulus strength of twice the diastolic threshold. Unipolar electrograms were filtered at 0.05–500 Hz and recorded at a sampling rate of 2,000 Hz (Bard Clearsign, CR Bard, NJ). Restitution curves were performed by pacing in three separate regions within the heart: the RV apex, the LVendo at the base, and the LVepi at the base, with recording made apico-basally in the RV and LV and transmurally at the base of the LV. Steady state pacing was performed at a basic cycle length of 600 ms for 3 min. An S1-S2 restitution protocol was performed, beginning with an extra stimulus (S2) at 1,000 ms. The S1-S2 coupling interval was then decremented in 50-ms steps until an S2 of 400 ms, then by 20-ms intervals between 300 and 400 ms, and thereafter in 5-ms steps, until effective refractory period (ERP) of the tissue. At ERP an S2 stimulus at 10 ms + ERP was applied followed by further decrementing S2 in steps of 2 ms to confirm ERP.

Data Analysis

Data analysis was conducted using a Matlab GUI that allows one to review and correct activation/repolarization markers measured automatically. A spectral estimate of the signal to noise ratio was obtained as the ratio between the power in the signal spectral band, defined between 0.5-40 Hz and in the noise spectral band, defined between 40-100 Hz. Signals with SNR lower than 13dB where not included in the analysis. At each S2 interval for every pacing location (RV, LV and CS), SECGTW markers (Figure 1. D) were assessed and compared with simultaneously recorded unipolar intracardiac repolarisation times (UEGMRT) in the LV and RV(Figure. 1C). In the unipolar contact electrograms, activation (AT) and repolarization times (RT) were measured at the minimum of the first derivative, min(dV/dt), of the signal within the depolarization phase and at the maximum of the first derivative, max(dV/dt), of the signal during the T-wave, respectively (Fig. 1C). The activation recovery interval, a standard surrogate of local APD, was measured as RT-AT (Fig. 1C). Dispersion of repolarization was computed as the interval between the minimum and maximum RT. For every beat ECG marker differences across individual leads and between leads were assessed for comparison of repolarization dispersion in the major anatomical axis. The axis assessed were apicobasal repolarization differences (measured the largest difference between apex minus base repolarization times with the heart), transmural dispersion of repolarization of the LV basal wall (measured as the largest difference between endocardial minus epicardial repolarization time), and right to left repolarization dispersion (measured as the largest difference of right and left repolarization times).

The SECGTW was analysed for time of onset of the T-wave (Ton), peak of the T-wave (Tpeak) and end of the Twave (Tend), in every ECG lead in every patient(Figure. 1D) at every cycle length. Tpeak was identified as the maximum of upright and the minimum of inverted T-waves, while Ton was localised as the local inflection point at the onset of the T-wave. Tend was calculated using the tangent method as the intersection between dv/dtmin in an upright T-wave or dv/dtmax in an inverted T-wave and the baseline (i.e.the intersection between the tangent to the latest flank of the T-wave and the baseline). This allowed comparison of T-wave duration, earlest Tiso to latest Tend, and differences between the upslope ends between different leads and intacardiac repolarization during a single beat across all 12 ECG leads and their association of repolarization dispersion in the major anatomical axes. Assessment of marker times differences within individual t-waves and across T-waves in the 12 lead ECG and their association with repolarization dispersion was performed using an automated R script (see statistical analysis section) that allowed subtraction of earliest and latest marker times between leads for each cycle length and displayed the best correlation for dispersion of repolarization in each major anatomical axis as shown in the results section.

In the following T-wave upslope corresponds to the ascending flank of a T-wave which in an upright Twave represents Ton-Tpeak and in an inverted T-wave represents Tpeak-Tend, “upslope end” refers to the end of the ascending flank of a T-wave, which corresponds to Tpeak in upright Twaves and to Tend in inverted T-waves. The interval between early and late upslope end was used as an estimate of repolarization dispersion. In total 23,946 individual SECGTW were analyzed and compared to regional UEGMRT, to assess the association of the SECGTW to the UEGMRT regardless of pacing cycle length and activation wavefront. All markers were measured with semi-automatic bespoke Matlab interface as in previous studies and manually corrected if needed. In total 23,946 individual SECGTW were analysed.

Statistics

Continuous variables are represented as mean ± SD if normally distributed and median (25th-75th quantile) if not normally distributed. Assessment of normality was assessed through histogram plots, Q-Q plots and the correspondence of the mean and median. Comparisons between measured intracardiac repolarization time and SECG markers were assessed using a paired T-test. Measurement similarity between SECG T-wave markers and the intracardiac T-wave were assessed by calculating the intraclass correlation coefficient (ICC), using a two way mixed model of absolute agreement. The relationship between the upslope of the T-wave on the SECG, regardless of polarity, and regional intracardiac repolarisation moments was assessed using sensitivity and specificity analysis, with sensitivity assessing whether regional repolarization occurs during the Twave upslope of the most proximal leads and specificity assesses whether repolarization of distant regions occurs outside the Twave upslope of the most proximal leads. Relationship between the dispersion of repolarization and measures within the ECG T-wave were assessed using ICC, and R2 of linear regression. A P value of ≤0.05 was considered statistically significant. Statistical analysis was performed using R statistical computing software (Version 3.2.2).
